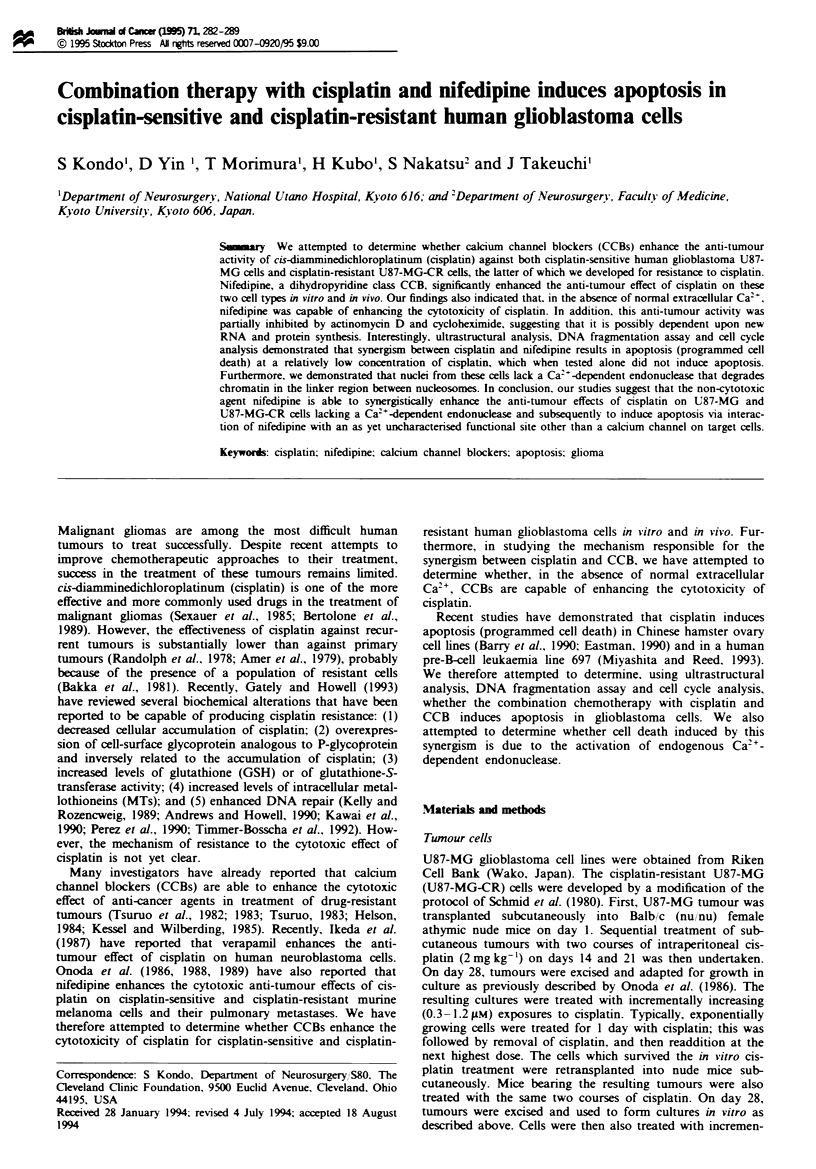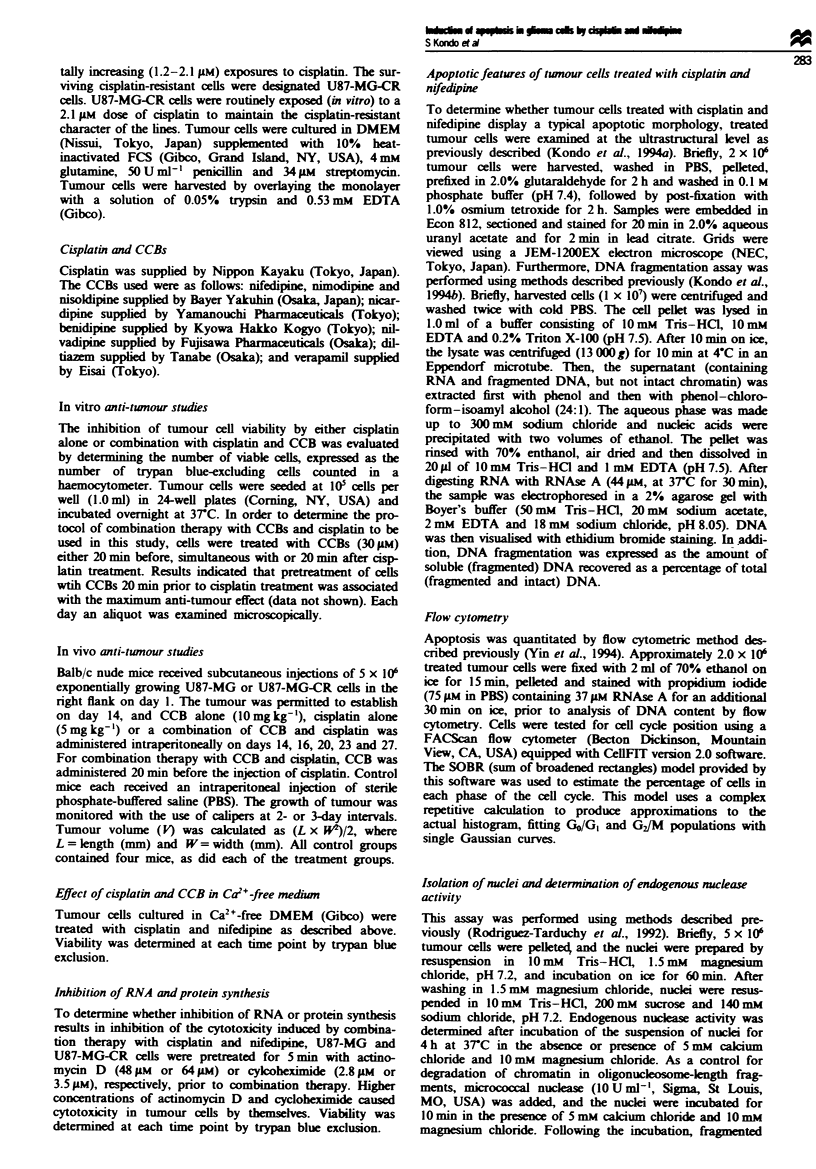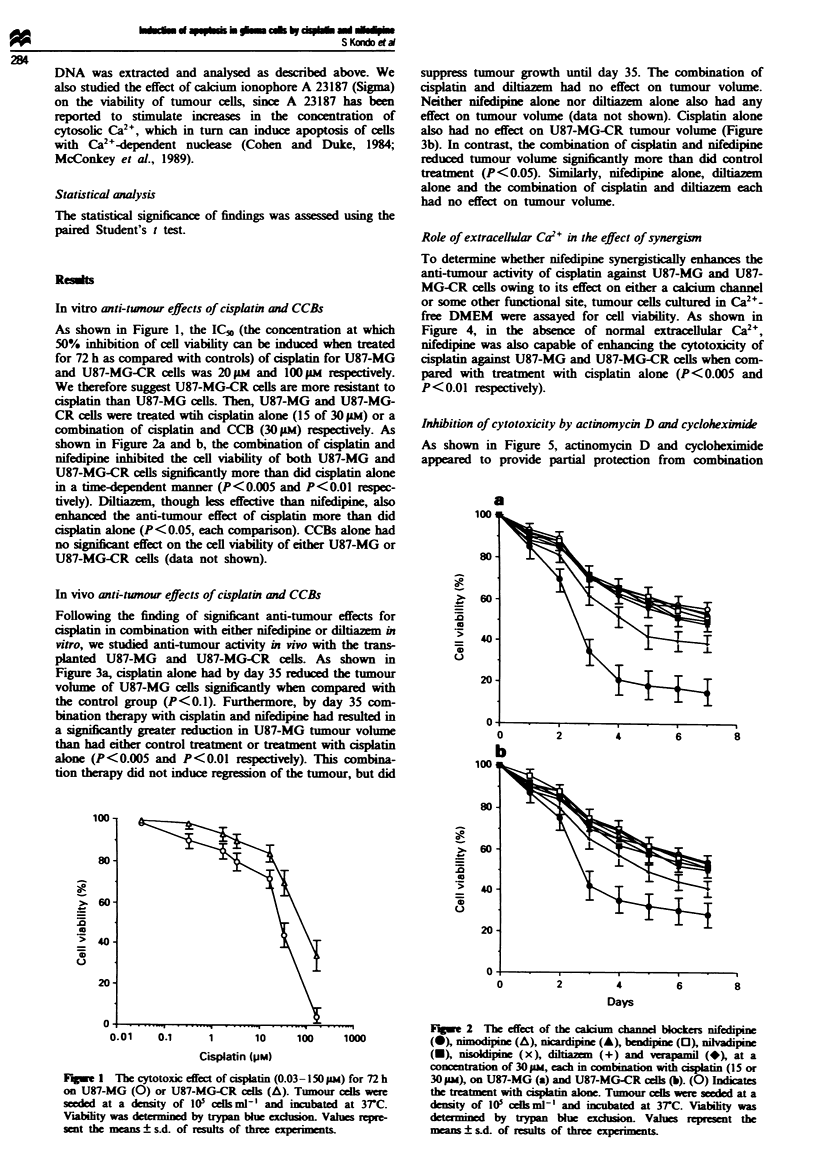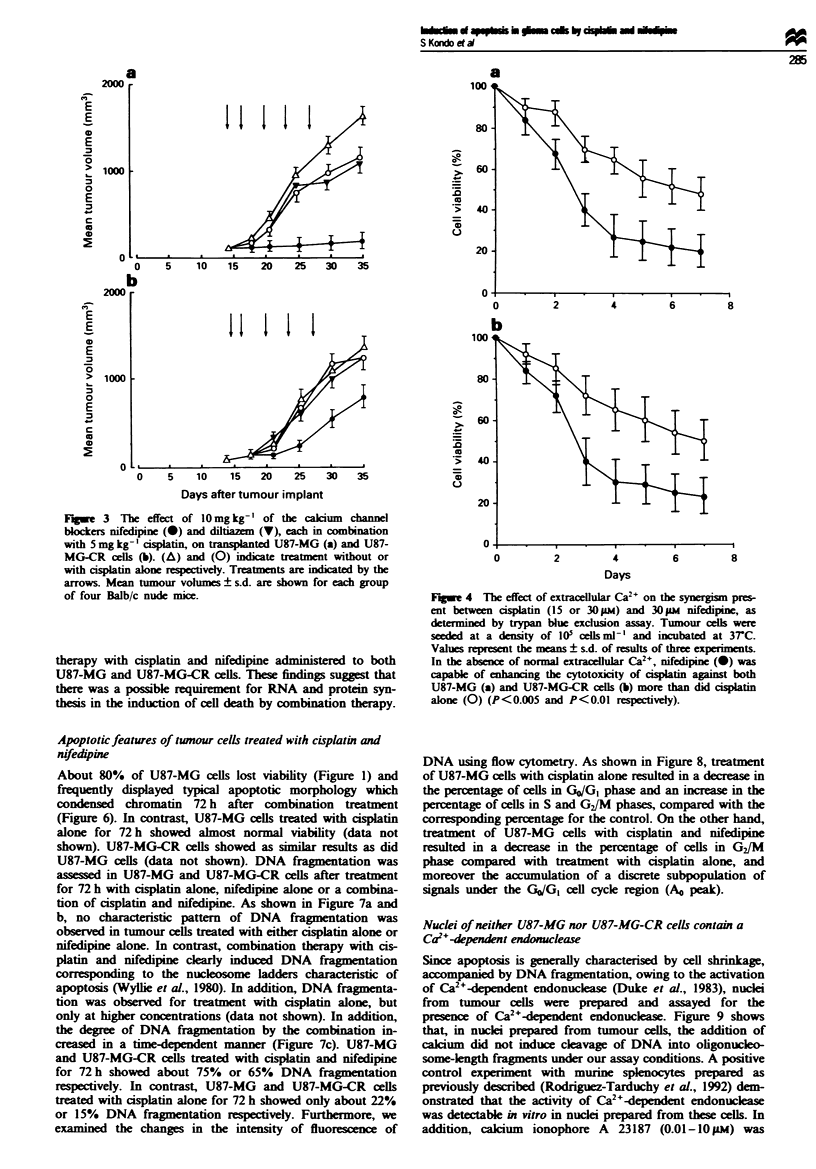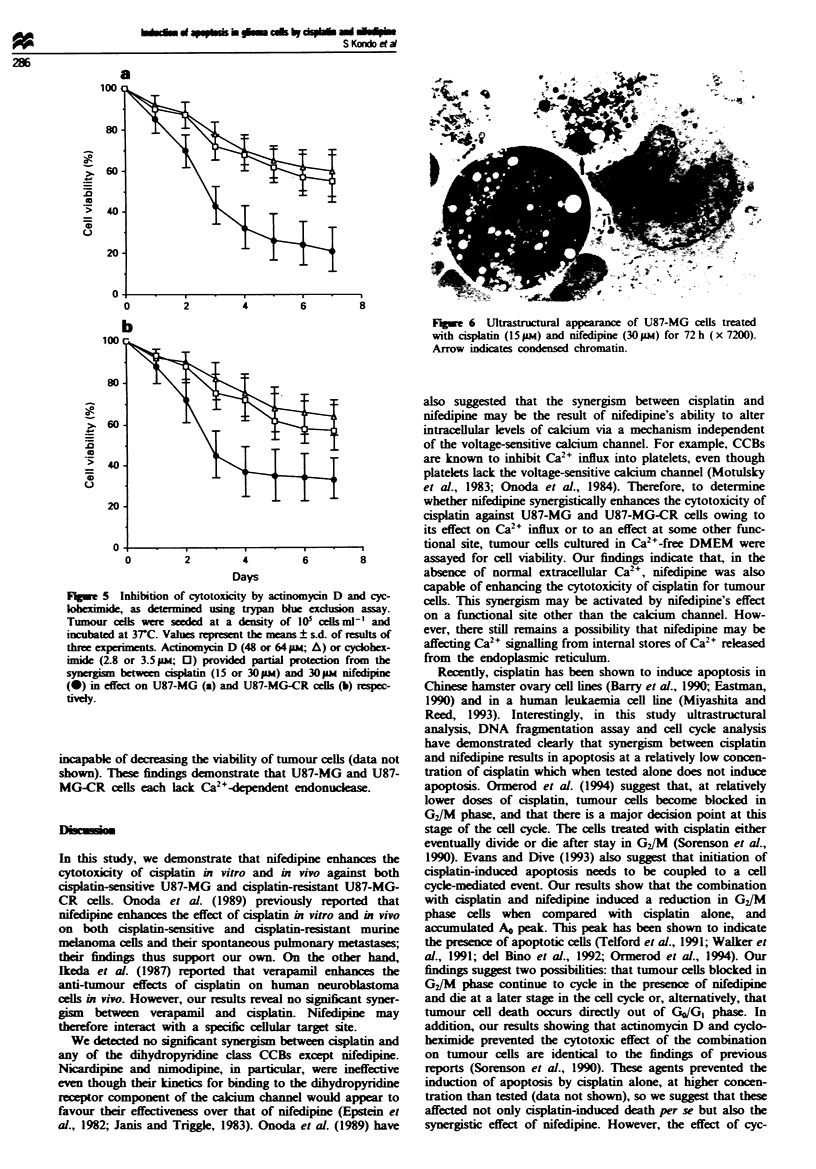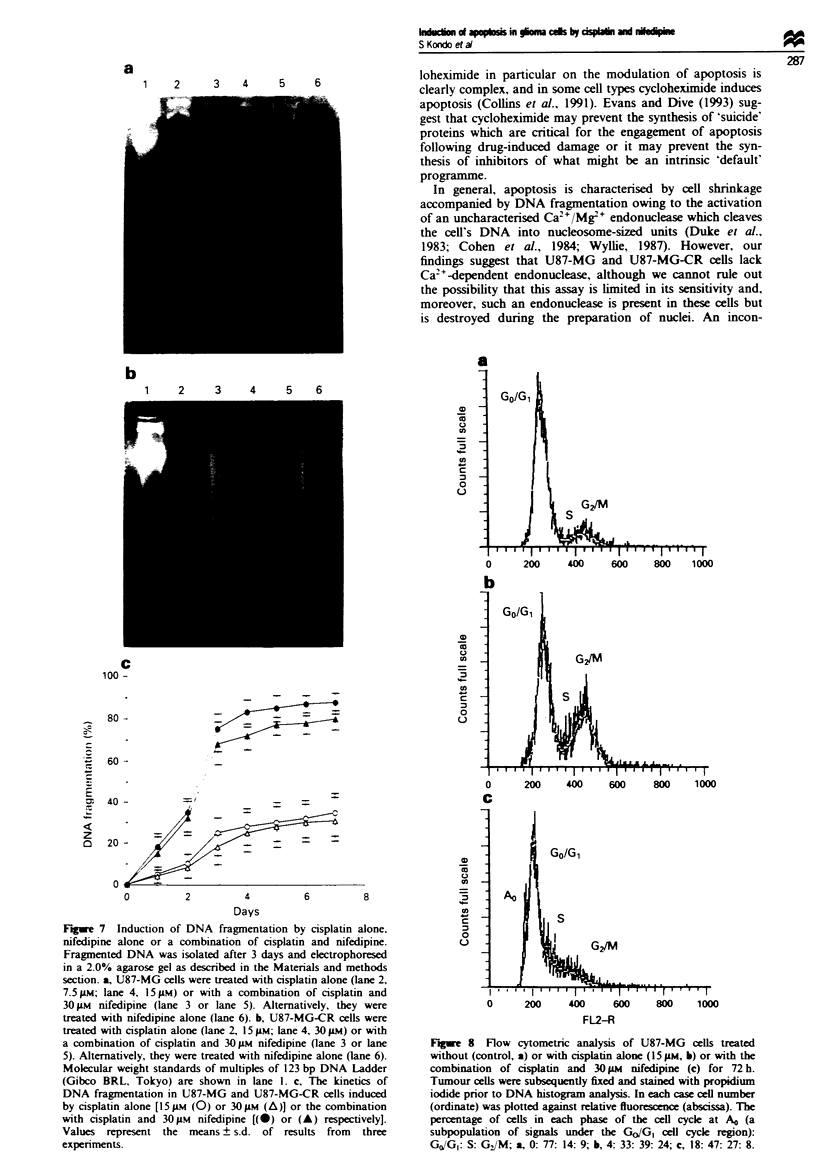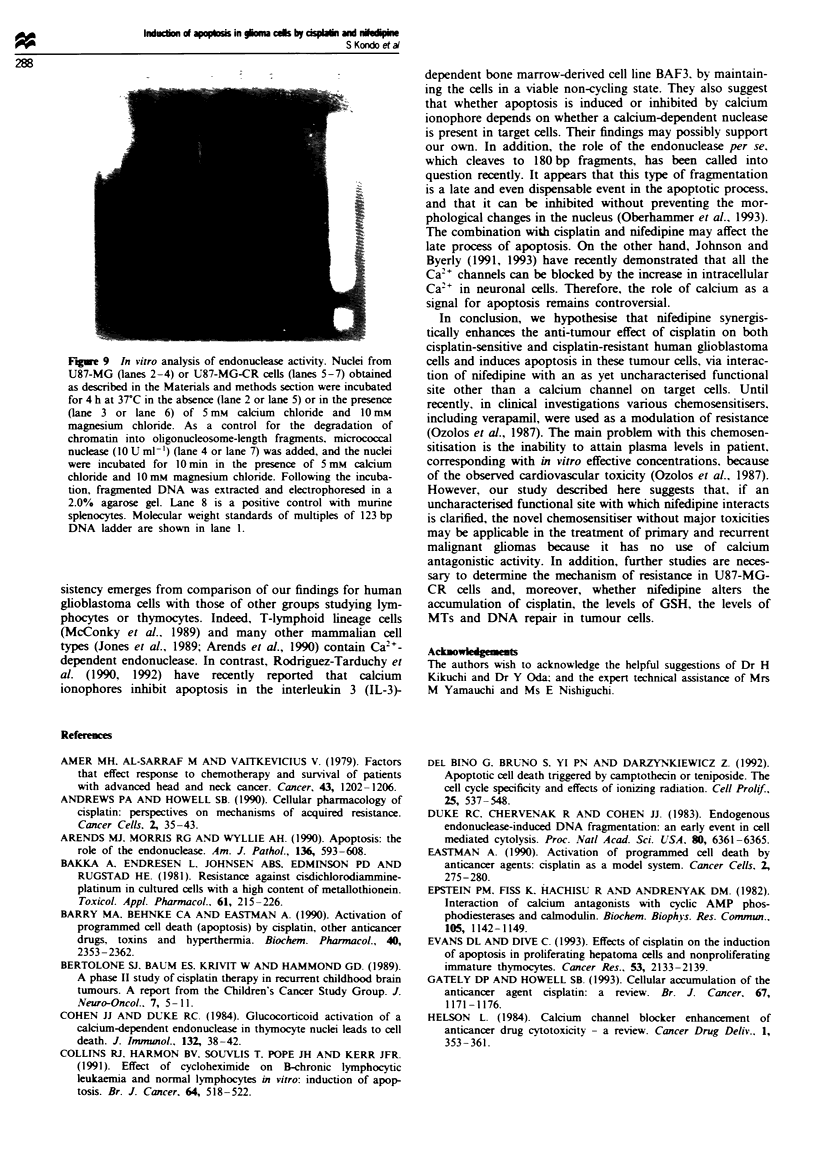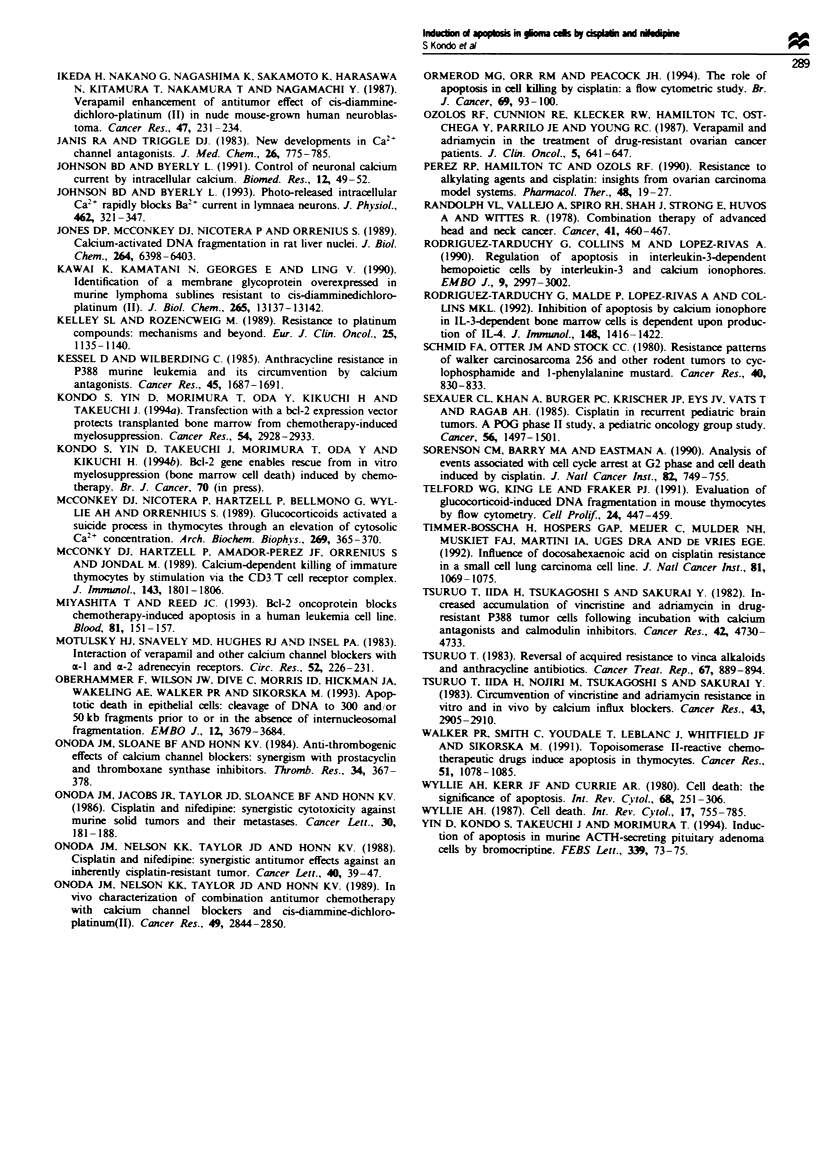# Combination therapy with cisplatin and nifedipine induces apoptosis in cisplatin-sensitive and cisplatin-resistant human glioblastoma cells.

**DOI:** 10.1038/bjc.1995.57

**Published:** 1995-02

**Authors:** S. Kondo, D. Yin, T. Morimura, H. Kubo, S. Nakatsu, J. Takeuchi

**Affiliations:** Department of Neurosurgery, National Utano Hospital, Kyoto, Japan.

## Abstract

**Images:**


British Journal of Cancer (1995) 71, 282-289

© 1995 Stockton Press All rights reserved 0007-0920/95 $9.00

### Combination therapy with cisplatin and nifedipine induces apoptosis in cisplatin-sensitive and cisplatin-resistant human glioblastoma cells

S Kondo[1], D Yin [1], T Morimura[1], H Kubo[1], S Nakatsu[2] and J Takeuchi[1]

[1]*Department of Neurosurgery, National Utano Hospital, Kyoto 616; and* [2]*Department of Neurosurgery, Faculty of Medicine, Kyoto University, Kyoto 606, Japan.*

**Summary** We attempted to determine whether calcium channel blockers (CCBs) enhance the anti-tumour activity of *cis*-diamminedichloroplatinum (cisplatin) against both cisplatin-sensitive human glioblastoma U87-MG cells and cisplatin-resistant U87-MG-CR cells, the latter of which we developed for resistance to cisplatin. Nifedipine, a dihydropyridine class CCB, significantly enhanced the anti-tumour effect of cisplatin on these two cell types *in vitro* and *in vivo*. Our findings also indicated that, in the absence of normal extracellular $Ca^{2+}$, nifedipine was capable of enhancing the cytotoxicity of cisplatin. In addition, this anti-tumour activity was partially inhibited by actinomycin D and cycloheximide, suggesting that it is possibly dependent upon new RNA and protein synthesis. Interestingly, ultrastructural analysis, DNA fragmentation assay and cell cycle analysis demonstrated that synergism between cisplatin and nifedipine results in apoptosis (programmed cell death) at a relatively low concentration of cisplatin, which when tested alone did not induce apoptosis. Furthermore, we demonstrated that nuclei from these cells lack a $Ca^{2+}$-dependent endonuclease that degrades chromatin in the linker region between nucleosomes. In conclusion, our studies suggest that the non-cytotoxic agent nifedipine is able to synergistically enhance the anti-tumour effects of cisplatin on U87-MG and U87-MG-CR cells lacking a $Ca^{2+}$-dependent endonuclease and subsequently to induce apoptosis via interaction of nifedipine with an as yet uncharacterised functional site other than a calcium channel on target cells.

**Keywords**: cisplatin; nifedipine; calcium channel blockers; apoptosis; glioma

Malignant gliomas are among the most difficult human tumours to treat successfully. Despite recent attempts to improve chemotherapeutic approaches to their treatment, success in the treatment of these tumours remains limited. *cis*-diamminedichloroplatinum (cisplatin) is one of the more effective and more commonly used drugs in the treatment of malignant gliomas (Sexauer *et al.*, 1985; Bertolone *et al.*, 1989). However, the effectiveness of cisplatin against recurrent tumours is substantially lower than against primary tumours (Randolph *et al.*, 1978; Amer *et al.*, 1979), probably because of the presence of a population of resistant cells (Bakka *et al.*, 1981). Recently, Gately and Howell (1993) have reviewed several biochemical alterations that have been reported to be capable of producing cisplatin resistance: (1) decreased cellular accumulation of cisplatin; (2) overexpression of cell-surface glycoprotein analogous to P-glycoprotein and inversely related to the accumulation of cisplatin; (3) increased levels of glutathione (GSH) or of glutathione-*S*-transferase activity; (4) increased levels of intracellular metallothioneins (MTs); and (5) enhanced DNA repair (Kelly and Rozencweig, 1989; Andrews and Howell, 1990; Kawai *et al.*, 1990; Perez *et al.*, 1990; Timmer-Bosscha *et al.*, 1992). However, the mechanism of resistance to the cytotoxic effect of cisplatin is not yet clear.

Many investigators have already reported that calcium channel blockers (CCBs) are able to enhance the cytotoxic effect of anti-cancer agents in treatment of drug-resistant tumours (Tsuruo *et al.*, 1982; 1983; Tsuruo, 1983; Helson, 1984; Kessel and Wilberding, 1985). Recently, Ikeda *et al.* (1987) have reported that verapamil enhances the anti-tumour effect of cisplatin on human neuroblastoma cells. Onoda *et al.* (1986, 1988, 1989) have also reported that nifedipine enhances the cytotoxic anti-tumour effects of cisplatin on cisplatin-sensitive and cisplatin-resistant murine melanoma cells and their pulmonary metastases. We have therefore attempted to determine whether CCBs enhance the cytotoxicity of cisplatin for cisplatin-sensitive and cisplatin-resistant human glioblastoma cells *in vitro* and *in vivo*. Furthermore, in studying the mechanism responsible for the synergism between cisplatin and CCB, we have attempted to determine whether, in the absence of normal extracellular $Ca^{2+}$, CCBs are capable of enhancing the cytotoxicity of cisplatin.

Recent studies have demonstrated that cisplatin induces apoptosis (programmed cell death) in Chinese hamster ovary cell lines (Barry *et al.*, 1990; Eastman, 1990) and in a human pre-B-cell leukaemia line 697 (Miyashita and Reed, 1993). We therefore attempted to determine, using ultrastructural analysis, DNA fragmentation assay and cell cycle analysis, whether the combination chemotherapy with cisplatin and CCB induces apoptosis in glioblastoma cells. We also attempted to determine whether cell death induced by this synergism is due to the activation of endogenous $Ca^{2+}$-dependent endonuclease.

### Materials and methods

### *Tumour cells*

U87-MG glioblastoma cell lines were obtained from Riken Cell Bank (Wako, Japan). The cisplatin-resistant U87-MG (U87-MG-CR) cells were developed by a modification of the protocol of Schmid *et al.* (1980). First, U87-MG tumour was transplanted subcutaneously into Balb c (nu nu) female athymic nude mice on day 1. Sequential treatment of subcutaneous tumours with two courses of intraperitoneal cisplatin ($2\ mg\ kg^{-1}$) on days 14 and 21 was then undertaken. On day 28, tumours were excised and adapted for growth in culture as previously described by Onoda *et al.* (1986). The resulting cultures were treated with incrementally increasing ($0.3-1.2\ \mu M$) exposures to cisplatin. Typically, exponentially growing cells were treated for 1 day with cisplatin; this was followed by removal of cisplatin, and then readdition at the next highest dose. The cells which survived the *in vitro* cisplatin treatment were retransplanted into nude mice subcutaneously. Mice bearing the resulting tumours were also treated with the same two courses of cisplatin. On day 28, tumours were excised and used to form cultures *in vitro* as described above. Cells were then also treated with incremen-

Correspondence: S Kondo, Department of Neurosurgery S80, The Cleveland Clinic Foundation, 9500 Euclid Avenue, Cleveland, Ohio 44195, USA
Received 28 January 1994; revised 4 July 1994; accepted 18 August 1994

tally increasing (1.2–2.1 μM) exposures to cisplatin. The surviving cisplatin-resistant cells were designated U87-MG-CR cells. U87-MG-CR cells were routinely exposed (*in vitro*) to a 2.1 μM dose of cisplatin to maintain the cisplatin-resistant character of the lines. Tumour cells were cultured in DMEM (Nissui, Tokyo, Japan) supplemented with 10% heat-inactivated FCS (Gibco, Grand Island, NY, USA), 4 mM glutamine, 50 U $ml^{-1}$ penicillin and 34 μM streptomycin. Tumour cells were harvested by overlaying the monolayer with a solution of 0.05% trypsin and 0.53 mM EDTA (Gibco).

*Cisplatin and CCBs*

Cisplatin was supplied by Nippon Kayaku (Tokyo, Japan). The CCBs used were as follows: nifedipine, nimodipine and nisoldipine supplied by Bayer Yakuhin (Osaka, Japan); nicardipine supplied by Yamanouchi Pharmaceuticals (Tokyo); benidipine supplied by Kyowa Hakko Kogyo (Tokyo); nilvadipine supplied by Fujisawa Pharmaceuticals (Osaka); diltiazem supplied by Tanabe (Osaka); and verapamil supplied by Eisai (Tokyo).

In vitro *anti-tumour studies*

The inhibition of tumour cell viability by either cisplatin alone or combination with cisplatin and CCB was evaluated by determining the number of viable cells, expressed as the number of trypan blue-excluding cells counted in a haemocytometer. Tumour cells were seeded at $10^5$ cells per well (1.0 ml) in 24-well plates (Corning, NY, USA) and incubated overnight at 37°C. In order to determine the protocol of combination therapy with CCBs and cisplatin to be used in this study, cells were treated with CCBs (30 μM) either 20 min before, simultaneous with or 20 min after cisplatin treatment. Results indicated that pretreatment of cells wtih CCBs 20 min prior to cisplatin treatment was associated with the maximum anti-tumour effect (data not shown). Each day an aliquot was examined microscopically.

In vivo *anti-tumour studies*

Balb c nude mice received subcutaneous injections of $5 \times 10^6$ exponentially growing U87-MG or U87-MG-CR cells in the right flank on day 1. The tumour was permitted to establish on day 14, and CCB alone (10 mg $kg^{-1}$), cisplatin alone (5 mg $kg^{-1}$) or a combination of CCB and cisplatin was administered intraperitoneally on days 14, 16, 20, 23 and 27. For combination therapy with CCB and cisplatin, CCB was administered 20 min before the injection of cisplatin. Control mice each received an intraperitoneal injection of sterile phosphate-buffered saline (PBS). The growth of tumour was monitored with the use of calipers at 2- or 3-day intervals. Tumour volume ($V$) was calculated as ($L \times W^2$) 2, where $L$ = length (mm) and $W$ = width (mm). All control groups contained four mice, as did each of the treatment groups.

*Effect of cisplatin and CCB in $Ca^{2+}$-free medium*

Tumour cells cultured in $Ca^{2+}$-free DMEM (Gibco) were treated with cisplatin and nifedipine as described above. Viability was determined at each time point by trypan blue exclusion.

*Inhibition of RNA and protein synthesis*

To determine whether inhibition of RNA or protein synthesis results in inhibition of the cytotoxicity induced by combination therapy with cisplatin and nifedipine, U87-MG and U87-MG-CR cells were pretreated for 5 min with actinomycin D (48 μM or 64 μM) or cylcoheximide (2.8 μM or 3.5 μM), respectively, prior to combination therapy. Higher concentrations of actinomycin D and cycloheximide caused cytotoxicity in tumour cells by themselves. Viability was determined at each time point by trypan blue exclusion.

*Apoptotic features of tumour cells treated with cisplatin and nifedipine*

To determine whether tumour cells treated with cisplatin and nifedipine display a typical apoptotic morphology, treated tumour cells were examined at the ultrastructural level as previously described (Kondo *et al.*, 1994*a*). Briefly, $2 \times 10^6$ tumour cells were harvested, washed in PBS, pelleted, prefixed in 2.0% glutaraldehyde for 2 h and washed in 0.1 M phosphate buffer (pH 7.4), followed by post-fixation with 1.0% osmium tetroxide for 2 h. Samples were embedded in Econ 812, sectioned and stained for 20 min in 2.0% aqueous uranyl acetate and for 2 min in lead citrate. Grids were viewed using a JEM-1200EX electron microscope (NEC, Tokyo, Japan). Furthermore, DNA fragmentation assay was performed using methods described previously (Kondo *et al.*, 1994*b*). Briefly, harvested cells ($1 \times 10^7$) were centrifuged and washed twice with cold PBS. The cell pellet was lysed in 1.0 ml of a buffer consisting of 10 mM Tris-HCl, 10 mM EDTA and 0.2% Triton X-100 (pH 7.5). After 10 min on ice, the lysate was centrifuged (13 000 ***g***) for 10 min at 4°C in an Eppendorf microtube. Then, the supernatant (containing RNA and fragmented DNA, but not intact chromatin) was extracted first with phenol and then with phenol-chloroform-isoamyl alcohol (24:1). The aqueous phase was made up to 300 mM sodium chloride and nucleic acids were precipitated with two volumes of ethanol. The pellet was rinsed with 70% enthanol, air dried and then dissolved in 20 μl of 10 mM Tris-HCl and 1 mM EDTA (pH 7.5). After digesting RNA with RNAse A (44 μM, at 37°C for 30 min), the sample was electrophoresed in a 2% agarose gel with Boyer's buffer (50 mM Tris-HCl, 20 mM sodium acetate, 2 mM EDTA and 18 mM sodium chloride, pH 8.05). DNA was then visualised with ethidium bromide staining. In addition, DNA fragmentation was expressed as the amount of soluble (fragmented) DNA recovered as a percentage of total (fragmented and intact) DNA.

*Flow cytometry*

Apoptosis was quantitated by flow cytometric method described previously (Yin *et al.*, 1994). Approximately $2.0 \times 10^6$ treated tumour cells were fixed with 2 ml of 70% ethanol on ice for 15 min, pelleted and stained with propidium iodide (75 μM in PBS) containing 37 μM RNAse A for an additional 30 min on ice, prior to analysis of DNA content by flow cytometry. Cells were tested for cell cycle position using a FACScan flow cytometer (Becton Dickinson, Mountain View, CA, USA) equipped with CellFIT version 2.0 software. The SOBR (sum of broadened rectangles) model provided by this software was used to estimate the percentage of cells in each phase of the cell cycle. This model uses a complex repetitive calculation to produce approximations to the actual histogram, fitting $G_0$ $G_1$ and $G_2$ M populations with single Gaussian curves.

*Isolation of nuclei and determination of endogenous nuclease activity*

This assay was performed using methods described previously (Rodriguez-Tarduchy *et al.*, 1992). Briefly, $5 \times 10^6$ tumour cells were pelleted and the nuclei were prepared by resuspension in 10 mM Tris-HCl, 1.5 mM magnesium chloride, pH 7.2, and incubation on ice for 60 min. After washing in 1.5 mM magnesium chloride, nuclei were resuspended in 10 mM Tris-HCl, 200 mM sucrose and 140 mM sodium chloride, pH 7.2. Endogenous nuclease activity was determined after incubation of the suspension of nuclei for 4 h at 37°C in the absence or presence of 5 mM calcium chloride and 10 mM magnesium chloride. As a control for degradation of chromatin in oligonucleosome-length fragments, micrococcal nuclease (10 U $ml^{-1}$, Sigma, St Louis, MO, USA) was added, and the nuclei were incubated for 10 min in the presence of 5 mM calcium chloride and 10 mM magnesium chloride. Following the incubation, fragmented

DNA was extracted and analysed as described above. We also studied the effect of calcium ionophore A 23187 (Sigma) on the viability of tumour cells, since A 23187 has been reported to stimulate increases in the concentration of cytosolic $Ca^{2+}$, which in turn can induce apoptosis of cells with $Ca^{2+}$-dependent nuclease (Cohen and Duke, 1984; McConkey *et al*., 1989).

*Statistical analysis*

The statistical significance of findings was assessed using the paired Student's *t* test.

### Results

In vitro *anti-tumour effects of cisplatin and CCBs*

As shown in Figure 1, the $IC_{50}$ (the concentration at which 50% inhibition of cell viability can be induced when treated for 72 h as compared with controls) of cisplatin for U87-MG and U87-MG-CR cells was 20 μM and 100 μM respectively. We therefore suggest U87-MG-CR cells are more resistant to cisplatin than U87-MG cells. Then, U87-MG and U87-MG-CR cells were treated wtih cisplatin alone (15 of 30 μM) or a combination of cisplatin and CCB (30 μM) respectively. As shown in Figure 2a and b, the combination of cisplatin and nifedipine inhibited the cell viability of both U87-MG and U87-MG-CR cells significantly more than did cisplatin alone in a time-dependent manner ($P<0.005$ and $P<0.01$ respectively). Diltiazem, though less effective than nifedipine, also enhanced the anti-tumour effect of cisplatin more than did cisplatin alone ($P<0.05$, each comparison). CCBs alone had no significant effect on the cell viability of either U87-MG or U87-MG-CR cells (data not shown).

In vivo *anti-tumour effects of cisplatin and CCBs*

Following the finding of significant anti-tumour effects for cisplatin in combination with either nifedipine or diltiazem *in vitro*, we studied anti-tumour activity *in vivo* with the transplanted U87-MG and U87-MG-CR cells. As shown in Figure 3a, cisplatin alone had by day 35 reduced the tumour volume of U87-MG cells significantly when compared with the control group ($P<0.1$). Furthermore, by day 35 combination therapy with cisplatin and nifedipine had resulted in a significantly greater reduction in U87-MG tumour volume than had either control treatment or treatment with cisplatin alone ($P<0.005$ and $P<0.01$ respectively). This combination therapy did not induce regression of the tumour, but did suppress tumour growth until day 35. The combination of cisplatin and diltiazem had no effect on tumour volume. Neither nifedipine alone nor diltiazem alone also had any effect on tumour volume (data not shown). Cisplatin alone also had no effect on U87-MG-CR tumour volume (Figure 3b). In contrast, the combination of cisplatin and nifedipine reduced tumour volume significantly more than did control treatment ($P<0.05$). Similarly, nifedipine alone, diltiazem alone and the combination of cisplatin and diltiazem each had no effect on tumour volume.

*Role of extracellular $Ca^{2+}$ in the effect of synergism*

To determine whether nifedipine synergistically enhances the anti-tumour activity of cisplatin against U87-MG and U87-MG-CR cells owing to its effect on either a calcium channel or some other functional site, tumour cells cultured in $Ca^{2+}$-free DMEM were assayed for cell viability. As shown in Figure 4, in the absence of normal extracellular $Ca^{2+}$, nifedipine was also capable of enhancing the cytotoxicity of cisplatin against U87-MG and U87-MG-CR cells when compared with treatment with cisplatin alone ($P<0.005$ and $P<0.01$ respectively).

*Inhibition of cytotoxicity by actinomycin D and cycloheximide*

As shown in Figure 5, actinomycin D and cycloheximide appeared to provide partial protection from combination

**Figure 1** The cytotoxic effect of cisplatin (0.03–150 μM) for 72 h on U87-MG (○) or U87-MG-CR cells (△). Tumour cells were seeded at a density of $10^5$ cells $ml^{-1}$ and incubated at 37°C. Viability was determined by trypan blue exclusion. Values represent the means ± s.d. of results of three experiments.

**Figure 2** The effect of the calcium channel blockers nifedipine (●), nimodipine (△), nicardipine (▲), bendipine (□), nilvadipine (■), nisoldipine (×), diltiazem (+) and verapamil (◆), at a concentration of 30 μM, each in combination with cisplatin (15 or 30 μM), on U87-MG (**a**) and U87-MG-CR cells (**b**). (○) Indicates the treatment with cisplatin alone. Tumour cells were seeded at a density of $10^5$ cells $ml^{-1}$ and incubated at 37°C. Viability was determined by trypan blue exclusion. Values represent the means ± s.d. of results of three experiments.

**Figure 3** The effect of $10\,mg\,kg^{-1}$ of the calcium channel blockers nifedipine (●) and diltiazem (▼), each in combination with $5\,mg\,kg^{-1}$ cisplatin, on transplanted U87-MG (**a**) and U87-MG-CR cells (**b**). (△) and (○) indicate treatment without or with cisplatin alone respectively. Treatments are indicated by the arrows. Mean tumour volumes ± s.d. are shown for each group of four Balb c nude mice.

**Figure 4** The effect of extracellular $Ca^{2+}$ on the synergism present between cisplatin (15 or 30 μM) and 30 μM nifedipine, as determined by trypan blue exclusion assay. Tumour cells were seeded at a density of $10^5$ cells $ml^{-1}$ and incubated at 37°C. Values represent the means ± s.d. of results of three experiments. In the absence of normal extracellular $Ca^{2+}$, nifedipine (●) was capable of enhancing the cytotoxicity of cisplatin against both U87-MG (**a**) and U87-MG-CR cells (**b**) more than did cisplatin alone (○) ($P < 0.005$ and $P < 0.01$ respectively).

therapy with cisplatin and nifedipine administered to both U87-MG and U87-MG-CR cells. These findings suggest that there was a possible requirement for RNA and protein synthesis in the induction of cell death by combination therapy.

*Apoptotic features of tumour cells treated with cisplatin and nifedipine*

About 80% of U87-MG cells lost viability (Figure 1) and frequently displayed typical apoptotic morphology which condensed chromatin 72 h after combination treatment (Figure 6). In contrast, U87-MG cells treated with cisplatin alone for 72 h showed almost normal viability (data not shown). U87-MG-CR cells showed as similar results as did U87-MG cells (data not shown). DNA fragmentation was assessed in U87-MG and U87-MG-CR cells after treatment for 72 h with cisplatin alone, nifedipine alone or a combination of cisplatin and nifedipine. As shown in Figure 7a and b, no characteristic pattern of DNA fragmentation was observed in tumour cells treated with either cisplatin alone or nifedipine alone. In contrast, combination therapy with cisplatin and nifedipine clearly induced DNA fragmentation corresponding to the nucleosome ladders characteristic of apoptosis (Wyllie *et al.*, 1980). In addition, DNA fragmentation was observed for treatment with cisplatin alone, but only at higher concentrations (data not shown). In addition, the degree of DNA fragmentation by the combination increased in a time-dependent manner (Figure 7c). U87-MG and U87-MG-CR cells treated with cisplatin and nifedipine for 72 h showed about 75% or 65% DNA fragmentation respectively. In contrast, U87-MG and U87-MG-CR cells treated with cisplatin alone for 72 h showed only about 22% or 15% DNA fragmentation respectively. Furthermore, we examined the changes in the intensity of fluorescence of DNA using flow cytometry. As shown in Figure 8, treatment of U87-MG cells with cisplatin alone resulted in a decrease in the percentage of cells in $G_0/G_1$ phase and an increase in the percentage of cells in S and $G_2/M$ phases, compared with the corresponding percentage for the control. On the other hand, treatment of U87-MG cells with cisplatin and nifedipine resulted in a decrease in the percentage of cells in $G_2/M$ phase compared with treatment with cisplatin alone, and moreover the accumulation of a discrete subpopulation of signals under the $G_0/G_1$ cell cycle region ($A_0$ peak).

*Nuclei of neither U87-MG nor U87-MG-CR cells contain a $Ca^{2+}$-dependent endonuclease*

Since apoptosis is generally characterised by cell shrinkage, accompanied by DNA fragmentation, owing to the activation of $Ca^{2+}$-dependent endonuclease (Duke *et al.*, 1983), nuclei from tumour cells were prepared and assayed for the presence of $Ca^{2+}$-dependent endonuclease. Figure 9 shows that, in nuclei prepared from tumour cells, the addition of calcium did not induce cleavage of DNA into oligonucleosome-length fragments under our assay conditions. A positive control experiment with murine splenocytes prepared as previously described (Rodriguez-Tarduchy *et al.*, 1992) demonstrated that the activity of $Ca^{2+}$-dependent endonuclease was detectable *in vitro* in nuclei prepared from these cells. In addition, calcium ionophore A 23187 (0.01–10 μM) was

**Figure 5** Inhibition of cytotoxicity by actinomycin D and cycloheximide, as determined using trypan blue exclusion assay. Tumour cells were seeded at a density of $10^5$ cells $ml^{-1}$ and incubated at 37°C. Values represent the means ± s.d. of results of three experiments. Actinomycin D (48 or 64 μM; △) or cycloheximide (2.8 or 3.5 μM; □) provided partial protection from the synergism between cisplatin (15 or 30 μM) and 30 μM nifedipine (●) in effect on U87-MG (**a**) and U87-MG-CR cells (**b**) respectively.

incapable of decreasing the viability of tumour cells (data not shown). These findings demonstrate that U87-MG and U87-MG-CR cells each lack $Ca^{2+}$-dependent endonuclease.

### Discussion

In this study, we demonstrate that nifedipine enhances the cytotoxicity of cisplatin *in vitro* and *in vivo* against both cisplatin-sensitive U87-MG and cisplatin-resistant U87-MG-CR cells. Onoda *et al.* (1989) previously reported that nifedipine enhances the effect of cisplatin *in vitro* and *in vivo* on both cisplatin-sensitive and cisplatin-resistant murine melanoma cells and their spontaneous pulmonary metastases; their findings thus support our own. On the other hand, Ikeda *et al.* (1987) reported that verapamil enhances the anti-tumour effects of cisplatin on human neuroblastoma cells *in vivo*. However, our results reveal no significant synergism between verapamil and cisplatin. Nifedipine may therefore interact with a specific cellular target site.

We detected no significant synergism between cisplatin and any of the dihydropyridine class CCBs except nifedipine. Nicardipine and nimodipine, in particular, were ineffective even though their kinetics for binding to the dihydropyridine receptor component of the calcium channel would appear to favour their effectiveness over that of nifedipine (Epstein *et al.*, 1982; Janis and Triggle, 1983). Onoda *et al.* (1989) have

**Figure 6** Ultrastructural appearance of U87-MG cells treated with cisplatin (15 μM) and nifedipine (30 μM) for 72 h (× 7200). Arrow indicates condensed chromatin.

also suggested that the synergism between cisplatin and nifedipine may be the result of nifedipine's ability to alter intracellular levels of calcium via a mechanism independent of the voltage-sensitive calcium channel. For example, CCBs are known to inhibit $Ca^{2+}$ influx into platelets, even though platelets lack the voltage-sensitive calcium channel (Motulsky *et al.*, 1983; Onoda *et al.*, 1984). Therefore, to determine whether nifedipine synergistically enhances the cytotoxicity of cisplatin against U87-MG and U87-MG-CR cells owing to its effect on $Ca^{2+}$ influx or to an effect at some other functional site, tumour cells cultured in $Ca^{2+}$-free DMEM were assayed for cell viability. Our findings indicate that, in the absence of normal extracellular $Ca^{2+}$, nifedipine was also capable of enhancing the cytotoxicity of cisplatin for tumour cells. This synergism may be activated by nifedipine's effect on a functional site other than the calcium channel. However, there still remains a possibility that nifedipine may be affecting $Ca^{2+}$ signalling from internal stores of $Ca^{2+}$ released from the endoplasmic reticulum.

Recently, cisplatin has been shown to induce apoptosis in Chinese hamster ovary cell lines (Barry *et al.*, 1990; Eastman, 1990) and in a human leukaemia cell line (Miyashita and Reed, 1993). Interestingly, in this study ultrastructural analysis, DNA fragmentation assay and cell cycle analysis have demonstrated clearly that synergism between cisplatin and nifedipine results in apoptosis at a relatively low concentration of cisplatin which when tested alone does not induce apoptosis. Ormerod *et al.* (1994) suggest that, at relatively lower doses of cisplatin, tumour cells become blocked in $G_2$ M phase, and that there is a major decision point at this stage of the cell cycle. The cells treated with cisplatin either eventually divide or die after stay in $G_2$ M (Sorenson *et al.*, 1990). Evans and Dive (1993) also suggest that initiation of cisplatin-induced apoptosis needs to be coupled to a cell cycle-mediated event. Our results show that the combination with cisplatin and nifedipine induced a reduction in $G_2$ M phase cells when compared with cisplatin alone, and accumulated $A_0$ peak. This peak has been shown to indicate the presence of apoptotic cells (Telford *et al.*, 1991; Walker *et al.*, 1991; del Bino *et al.*, 1992; Ormerod *et al.*, 1994). Our findings suggest two possibilities: that tumour cells blocked in $G_2$ M phase continue to cycle in the presence of nifedipine and die at a later stage in the cell cycle or, alternatively, that tumour cell death occurs directly out of $G_0$ $G_1$ phase. In addition, our results showing that actinomycin D and cycloheximide prevented the cytotoxic effect of the combination on tumour cells are identical to the findings of previous reports (Sorenson *et al.*, 1990). These agents prevented the induction of apoptosis by cisplatin alone, at higher concentration than tested (data not shown), so we suggest that these affected not only cisplatin-induced death *per se* but also the synergistic effect of nifedipine. However, the effect of cyc-

**Figure 7** Induction of DNA fragmentation by cisplatin alone, nifedipine alone or a combination of cisplatin and nifedipine. Fragmented DNA was isolated after 3 days and electrophoresed in a 2.0% agarose gel as described in the Materials and methods section. **a**, U87-MG cells were treated with cisplatin alone (lane 2, 7.5 μM; lane 4, 15 μM) or with a combination of cisplatin and 30 μM nifedipine (lane 3 or lane 5). Alternatively, they were treated with nifedipine alone (lane 6). **b**, U87-MG-CR cells were treated with cisplatin alone (lane 2, 15 μM; lane 4, 30 μM) or with a combination of cisplatin and 30 μM nifedipine (lane 3 or lane 5). Alternatively, they were treated with nifedipine alone (lane 6). Molecular weight standards of multiples of 123 bp DNA Ladder (Gibco BRL, Tokyo) are shown in lane 1. **c**, The kinetics of DNA fragmentation in U87-MG and U87-MG-CR cells induced by cisplatin alone [15 μM (○) or 30 μM (△)] or the combination with cisplatin and 30 μM nifedipine [(●) or (▲) respectively]. Values represent the means ± s.d. of results from three experiments.

loheximide in particular on the modulation of apoptosis is clearly complex, and in some cell types cycloheximide induces apoptosis (Collins *et al.*, 1991). Evans and Dive (1993) suggest that cycloheximide may prevent the synthesis of 'suicide' proteins which are critical for the engagement of apoptosis following drug-induced damage or it may prevent the synthesis of inhibitors of what might be an intrinsic 'default' programme.

In general, apoptosis is characterised by cell shrinkage accompanied by DNA fragmentation owing to the activation of an uncharacterised $Ca^{2+}/Mg^{2+}$ endonuclease which cleaves the cell's DNA into nucleosome-sized units (Duke *et al.*, 1983; Cohen *et al.*, 1984; Wyllie, 1987). However, our findings suggest that U87-MG and U87-MG-CR cells lack $Ca^{2+}$-dependent endonuclease, although we cannot rule out the possibility that this assay is limited in its sensitivity and, moreover, such an endonuclease is present in these cells but is destroyed during the preparation of nuclei. An incon-

**Figure 8** Flow cytometric analysis of U87-MG cells treated without (control, **a**) or with cisplatin alone (15 μM, **b**) or with the combination of cisplatin and 30 μM nifedipine (**c**) for 72 h. Tumour cells were subsequently fixed and stained with propidium iodide prior to DNA histogram analysis. In each case cell number (ordinate) was plotted against relative fluorescence (abscissa). The percentage of cells in each phase of the cell cycle at $A_0$ (a subpopulation of signals under the $G_0/G_1$ cell cycle region): $G_0/G_1$: S: $G_2/M$; **a**, 0: 77: 14: 9; **b**, 4: 33: 39: 24; **c**, 18: 47: 27: 8.

**Figure 9** *In vitro* analysis of endonuclease activity. Nuclei from U87-MG (lanes 2–4) or U87-MG-CR cells (lanes 5–7) obtained as described in the Materials and methods section were incubated for 4 h at 37°C in the absence (lane 2 or lane 5) or in the presence (lane 3 or lane 6) of 5 mM calcium chloride and 10 mM magnesium chloride. As a control for the degradation of chromatin into oligonucleosome-length fragments, micrococcal nuclease (10 U $ml^{-1}$) (lane 4 or lane 7) was added, and the nuclei were incubated for 10 min in the presence of 5 mM calcium chloride and 10 mM magnesium chloride. Following the incubation, fragmented DNA was extracted and electrophoresed in a 2.0% agarose gel. Lane 8 is a positive control with murine splenocytes. Molecular weight standards of multiples of 123 bp DNA ladder are shown in lane 1.

sistency emerges from comparison of our findings for human glioblastoma cells with those of other groups studying lymphocytes or thymocytes. Indeed, T-lymphoid lineage cells (McConky *et al.*, 1989) and many other mammalian cell types (Jones *et al.*, 1989; Arends *et al.*, 1990) contain $Ca^{2+}$-dependent endonuclease. In contrast, Rodriguez-Tarduchy *et al.* (1990, 1992) have recently reported that calcium ionophores inhibit apoptosis in the interleukin 3 (IL-3)-dependent bone marrow-derived cell line BAF3, by maintaining the cells in a viable non-cycling state. They also suggest that whether apoptosis is induced or inhibited by calcium ionophore depends on whether a calcium-dependent nuclease is present in target cells. Their findings may possibly support our own. In addition, the role of the endonuclease *per se*, which cleaves to 180 bp fragments, has been called into question recently. It appears that this type of fragmentation is a late and even dispensable event in the apoptotic process, and that it can be inhibited without preventing the morphological changes in the nucleus (Oberhammer *et al.*, 1993). The combination with cisplatin and nifedipine may affect the late process of apoptosis. On the other hand, Johnson and Byerly (1991, 1993) have recently demonstrated that all the $Ca^{2+}$ channels can be blocked by the increase in intracellular $Ca^{2+}$ in neuronal cells. Therefore, the role of calcium as a signal for apoptosis remains controversial.

In conclusion, we hypothesise that nifedipine synergistically enhances the anti-tumour effect of cisplatin on both cisplatin-sensitive and cisplatin-resistant human glioblastoma cells and induces apoptosis in these tumour cells, via interaction of nifedipine with an as yet uncharacterised functional site other than a calcium channel on target cells. Until recently, in clinical investigations various chemosensitisers, including verapamil, were used as a modulation of resistance (Ozolos *et al.*, 1987). The main problem with this chemosensitisation is the inability to attain plasma levels in patient, corresponding with *in vitro* effective concentrations, because of the observed cardiovascular toxicity (Ozolos *et al.*, 1987). However, our study described here suggests that, if an uncharacterised functional site with which nifedipine interacts is clarified, the novel chemosensitiser without major toxicities may be applicable in the treatment of primary and recurrent malignant gliomas because it has no use of calcium antagonistic activity. In addition, further studies are necessary to determine the mechanism of resistance in U87-MG-CR cells and, moreover, whether nifedipine alters the accumulation of cisplatin, the levels of GSH, the levels of MTs and DNA repair in tumour cells.

**Acknowledgements**
The authors wish to acknowledge the helpful suggestions of Dr H Kikuchi and Dr Y Oda; and the expert technical assistance of Mrs M Yamauchi and Ms E Nishiguchi.

**References**

AMER MH, AL-SARRAF M AND VAITKEVICIUS V. (1979). Factors that effect response to chemotherapy and survival of patients with advanced head and neck cancer. *Cancer*, **43**, 1202–1206.

ANDREWS PA AND HOWELL SB. (1990). Cellular pharmacology of cisplatin: perspectives on mechanisms of acquired resistance. *Cancer Cells*, **2**, 35–43.

ARENDS MJ, MORRIS RG AND WYLLIE AH. (1990). Apoptosis: the role of the endonuclease. *Am. J. Pathol.*, **136**, 593–608.

BAKKA A, ENDRESEN L, JOHNSEN ABS, EDMINSON PD AND RUGSTAD HE. (1981). Resistance against cisdichlorodiammine-platinum in cultured cells with a high content of metallothionein. *Toxicol. Appl. Pharmacol.*, **61**, 215–226.

BARRY MA, BEHNKE CA AND EASTMAN A. (1990). Activation of programmed cell death (apoptosis) by cisplatin, other anticancer drugs, toxins and hyperthermia. *Biochem. Pharmacol.*, **40**, 2353–2362.

BERTOLONE SJ, BAUM ES, KRIVIT W AND HAMMOND GD. (1989). A phase II study of cisplatin therapy in recurrent childhood brain tumours. A report from the Children's Cancer Study Group. *J. Neuro-Oncol.*, **7**, 5–11.

COHEN JJ AND DUKE RC. (1984). Glucocorticoid activation of a calcium-dependent endonuclease in thymocyte nuclei leads to cell death. *J. Immunol.*, **132**, 38–42.

COLLINS RJ, HARMON BV, SOUVLIS T, POPE JH AND KERR JFR. (1991). Effect of cycloheximide on B-chronic lymphocytic leukaemia and normal lymphocytes *in vitro*: induction of apoptosis. *Br. J. Cancer*, **64**, 518–522.

DEL BINO G, BRUNO S, YI PN AND DARZYNKIEWICZ Z. (1992). Apoptotic cell death triggered by camptothecin or teniposide. The cell cycle specificity and effects of ionizing radiation. *Cell Prolif.*, **25**, 537–548.

DUKE RC, CHERVENAK R AND COHEN JJ. (1983). Endogenous endonuclease-induced DNA fragmentation: an early event in cell mediated cytolysis. *Proc. Natl Acad. Sci. USA*, **80**, 6361–6365.

EASTMAN A. (1990). Activation of programmed cell death by anticancer agents: cisplatin as a model system. *Cancer Cells*, **2**, 275–280.

EPSTEIN PM, FISS K, HACHISU R AND ANDRENYAK DM. (1982). Interaction of calcium antagonists with cyclic AMP phosphodiesterases and calmodulin. *Biochem. Biophys. Res. Commun.*, **105**, 1142–1149.

EVANS DL AND DIVE C. (1993). Effects of cisplatin on the induction of apoptosis in proliferating hepatoma cells and nonproliferating immature thymocytes. *Cancer Res.*, **53**, 2133–2139.

GATELY DP AND HOWELL SB. (1993). Cellular accumulation of the anticancer agent cisplatin: a review. *Br. J. Cancer*, **67**, 1171–1176.

HELSON L. (1984). Calcium channel blocker enhancement of anticancer drug cytotoxicity – a review. *Cancer Drug Deliv.*, **1**, 353–361.

IKEDA H. NAKANO G. NAGASHIMA K. SAKAMOTO K. HARASAWA N. KITAMURA T. NAKAMURA T AND NAGAMACHI Y. (1987). Verapamil enhancement of antitumor effect of cis-diamminedichloro-platinum (II) in nude mouse-grown human neuroblastoma. *Cancer Res.*, **47**, 231-234.

JANIS RA AND TRIGGLE DJ. (1983). New developments in $Ca^{2+}$ channel antagonists. *J. Med. Chem.*, **26**, 775-785.

JOHNSON BD AND BYERLY L. (1991). Control of neuronal calcium current by intracellular calcium. *Biomed. Res.*, **12**, 49-52.

JOHNSON BD AND BYERLY L. (1993). Photo-released intracellular $Ca^{2+}$ rapidly blocks $Ba^{2+}$ current in lymnaea neurons. *J. Physiol.*, **462**, 321-347.

JONES DP. MCCONKEY DJ. NICOTERA P AND ORRENIUS S. (1989). Calcium-activated DNA fragmentation in rat liver nuclei. *J. Biol. Chem.*, **264**, 6398-6403.

KAWAI K. KAMATANI N. GEORGES E AND LING V. (1990). Identification of a membrane glycoprotein overexpressed in murine lymphoma sublines resistant to cis-diamminedichloroplatinum (II). *J. Biol. Chem.*, **265**, 13137-13142.

KELLEY SL AND ROZENCWEIG M. (1989). Resistance to platinum compounds: mechanisms and beyond. *Eur. J. Clin. Oncol.*, **25**, 1135-1140.

KESSEL D AND WILBERDING C. (1985). Anthracycline resistance in P388 murine leukemia and its circumvention by calcium antagonists. *Cancer Res.*, **45**, 1687-1691.

KONDO S. YIN D. MORIMURA T. ODA Y. KIKUCHI H AND TAKEUCHI J. (1994*a*). Transfection with a bcl-2 expression vector protects transplanted bone marrow from chemotherapy-induced myelosuppression. *Cancer Res.*, **54**, 2928-2933.

KONDO S. YIN D. TAKEUCHI J. MORIMURA T. ODA Y AND KIKUCHI H. (1994*b*). Bcl-2 gene enables rescue from in vitro myelosuppression (bone marrow cell death) induced by chemotherapy. *Br. J. Cancer*, **70** (in press).

MCCONKEY DJ. NICOTERA P. HARTZELL P. BELLMONO G. WYLLIE AH AND ORRENHIUS S. (1989). Glucocorticoids activated a suicide process in thymocytes through an elevation of cytosolic $Ca^{2+}$ concentration. *Arch. Biochem. Biophys.*, **269**, 365-370.

MCCONKY DJ. HARTZELL P. AMADOR-PEREZ JF. ORRENIUS S AND JONDAL M. (1989). Calcium-dependent killing of immature thymocytes by stimulation via the CD3 T cell receptor complex. *J. Immunol.*, **143**, 1801-1806.

MIYASHITA T AND REED JC. (1993). Bcl-2 oncoprotein blocks chemotherapy-induced apoptosis in a human leukemia cell line. *Blood*, **81**, 151-157.

MOTULSKY HJ. SNAVELY MD. HUGHES RJ AND INSEL PA. (1983). Interaction of verapamil and other calcium channel blockers with α-1 and α-2 adrenecyin receptors. *Circ. Res.*, **52**, 226-231.

OBERHAMMER F. WILSON JW. DIVE C. MORRIS ID. HICKMAN JA. WAKELING AE. WALKER PR AND SIKORSKA M. (1993). Apoptotic death in epithelial cells: cleavage of DNA to 300 and or 50 kb fragments prior to or in the absence of internucleosomal fragmentation. *EMBO J.*, **12**, 3679-3684.

ONODA JM. SLOANE BF AND HONN KV. (1984). Anti-thrombogenic effects of calcium channel blockers: synergism with prostacyclin and thromboxane synthase inhibitors. *Thromb. Res.*, **34**, 367-378.

ONODA JM. JACOBS JR. TAYLOR JD. SLOANCE BF AND HONN KV. (1986). Cisplatin and nifedipine: synergistic cytotoxicity against murine solid tumors and their metastases. *Cancer Lett.*, **30**, 181-188.

ONODA JM. NELSON KK. TAYLOR JD AND HONN KV. (1988). Cisplatin and nifedipine: synergistic antitumor effects against an inherently cisplatin-resistant tumor. *Cancer Lett.*, **40**, 39-47.

ONODA JM. NELSON KK. TAYLOR JD AND HONN KV. (1989). In vivo characterization of combination antitumor chemotherapy with calcium channel blockers and cis-diammine-dichloroplatinum(II). *Cancer Res.*, **49**, 2844-2850.

ORMEROD MG. ORR RM AND PEACOCK JH. (1994). The role of apoptosis in cell killing by cisplatin: a flow cytometric study. *Br. J. Cancer*, **69**, 93-100.

OZOLOS RF. CUNNION RE. KLECKER RW. HAMILTON TC. OSTCHEGA Y. PARRILO JE AND YOUNG RC. (1987). Verapamil and adriamycin in the treatment of drug-resistant ovarian cancer patients. *J. Clin. Oncol.*, **5**, 641-647.

PEREZ RP. HAMILTON TC AND OZOLS RF. (1990). Resistance to alkylating agents and cisplatin: insights from ovarian carcinoma model systems. *Pharmacol. Ther.*, **48**, 19-27.

RANDOLPH VL. VALLEJO A. SPIRO RH. SHAH J. STRONG E. HUVOS A AND WITTES R. (1978). Combination therapy of advanced head and neck cancer. *Cancer*, **41**, 460-467.

RODRIGUEZ-TARDUCHY G. COLLINS M AND LOPEZ-RIVAS A. (1990). Regulation of apoptosis in interleukin-3-dependent hemopoietic cells by interleukin-3 and calcium ionophores. *EMBO J.*, **9**, 2997-3002.

RODRIGUEZ-TARDUCHY G. MALDE P. LOPEZ-RIVAS A AND COLLINS MKL. (1992). Inhibition of apoptosis by calcium ionophore in IL-3-dependent bone marrow cells is dependent upon production of IL-4. *J. Immunol.*, **148**, 1416-1422.

SCHMID FA. OTTER JM AND STOCK CC. (1980). Resistance patterns of walker carcinosarcoma 256 and other rodent tumors to cyclophosphamide and l-phenylalanine mustard. *Cancer Res.*, **40**, 830-833.

SEXAUER CL. KHAN A. BURGER PC. KRISCHER JP. EYS JV. VATS T AND RAGAB AH. (1985). Cisplatin in recurrent pediatric brain tumors. A POG phase II study, a pediatric oncology group study. *Cancer*, **56**, 1497-1501.

SORENSON CM. BARRY MA AND EASTMAN A. (1990). Analysis of events associated with cell cycle arrest at G2 phase and cell death induced by cisplatin. *J. Natl Cancer Inst.*, **82**, 749-755.

TELFORD WG. KING LE AND FRAKER PJ. (1991). Evaluation of glucocorticoid-induced DNA fragmentation in mouse thymocytes by flow cytometry. *Cell Prolif.*, **24**, 447-459.

TIMMER-BOSSCHA H. HOSPERS GAP. MEIJER C. MULDER NH. MUSKIET FAJ. MARTINI IA. UGES DRA AND DE VRIES EGE. (1992). Influence of docosahexaenoic acid on cisplatin resistance in a small cell lung carcinoma cell line. *J. Natl Cancer Inst.*, **81**, 1069-1075.

TSURUO T. IIDA H. TSUKAGOSHI S AND SAKURAI Y. (1982). Increased accumulation of vincristine and adriamycin in drug-resistant P388 tumor cells following incubation with calcium antagonists and calmodulin inhibitors. *Cancer Res.*, **42**, 4730-4733.

TSURUO T. (1983). Reversal of acquired resistance to vinca alkaloids and anthracycline antibiotics. *Cancer Treat. Rep.*, **67**, 889-894.

TSURUO T. IIDA H. NOJIRI M. TSUKAGOSHI S AND SAKURAI Y. (1983). Circumvention of vincristine and adriamycin resistance in vitro and in vivo by calcium influx blockers. *Cancer Res.*, **43**, 2905-2910.

WALKER PR. SMITH C. YOUDALE T. LEBLANC J. WHITFIELD JF AND SIKORSKA M. (1991). Topoisomerase II-reactive chemotherapeutic drugs induce apoptosis in thymocytes. *Cancer Res.*, **51**, 1078-1085.

WYLLIE AH. KERR JF AND CURRIE AR. (1980). Cell death: the significance of apoptosis. *Int. Rev. Cytol.*, **68**, 251-306.

WYLLIE AH. (1987). Cell death. *Int. Rev. Cytol.*, **17**, 755-785.

YIN D. KONDO S. TAKEUCHI J AND MORIMURA T. (1994). Induction of apoptosis in murine ACTH-secreting pituitary adenoma cells by bromocriptine. *FEBS Lett.*, **339**, 73-75.